# The relationship between truncation and phosphorylation at the C-terminus of tau protein in the paired helical filaments of Alzheimer's disease

**DOI:** 10.3389/fnins.2015.00033

**Published:** 2015-02-11

**Authors:** Paola Flores-Rodríguez, Miguel A. Ontiveros-Torres, María C. Cárdenas-Aguayo, Juan P. Luna-Arias, Marco A. Meraz-Ríos, Amparo Viramontes-Pintos, Charles R. Harrington, Claude M. Wischik, Raúl Mena, Benjamin Florán-Garduño, José Luna-Muñoz

**Affiliations:** ^1^Departments of Physiology, Biophysics, and Neurosciences, Centro de Investigación y de Estudios Avanzados del IPN (Instituto Politecnico Nacional)Gustavo A. Madero, Mexico; ^2^Departments of Cell Biology, Centro de Investigación y de Estudios Avanzados del IPN (Instituto Politecnico Nacional)Gustavo A. Madero, Mexico; ^3^Departments of Molecular Biomedicine, Centro de Investigación y de Estudios Avanzados del IPN (Instituto Politecnico Nacional)Gustavo A. Madero, Mexico; ^4^Brain Bank-Laboratorio Nacional de Servicios Experimentales, Department of National Laboratories of Experimental Services, Centro de Investigación y de Estudios Avanzados del Instituto Politecnico NacionalGustavo A. Madero, Mexico; ^5^School of Medicine and Dentistry, University of AberdeenAberdeen, UK

**Keywords:** tau protein, truncation, neurotoxicity, neurofibrillary tangles, PHFs, tau oligomers, Alzheimer's disease

## Abstract

We previously demonstrated that, in the early stages of tau processing in Alzheimer's disease, the N-terminal part of the molecule undergoes a characteristic cascade of phosphorylation and progressive misfolding of the proteins resulting in a structural conformation detected by Alz-50. In this immunohistochemical study of AD brain tissue, we have found that C-terminal truncation of tau at Asp-421 was an early event in tau aggregation and analyzed the relationship between phospho-dependent tau epitopes located at the C-terminus with truncation at Glu-391. The aim of this study was to determine whether C-terminal truncation may trigger events leading to the assembly of insoluble PHFs from soluble tau aggregates present in pre-tangle cells. Our findings suggest that there is a complex interaction between phosphorylated and truncated tau species. A model is presented here in which truncated tau protein represents an early neurotoxic species while phosphorylated tau species may provide a neuroprotective role in Alzheimer's disease.

## Introduction

Tau pathology, a principal hallmark of Alzheimer's disease, is characterized by abnormal hyperphosphorylation and truncation of tau proteins (Wischik et al., 1988a; Goedert et al., 1992; Novak et al., 1993; Mena et al., 1996). Both molecular events are associated with the formation of paired helical filaments (PHFs) and the appearance of the neurofibrillary tangles (NFTs) within the cytoplasm of vulnerable cells (Wischik et al., 1988b; Novak et al., 1993; Mena et al., 1995, 1996; Guillozet-Bongaarts et al., 2005; Luna-Munoz et al., 2007). Although it has been suggested that phosphorylation of tau is associated with PHF assembly, evidence to confirm this has not been substantiated, since these studies have been based upon *in vitro* experiments and animal models rather than Alzheimer's disease tissue (Alonso et al., 1996, 2001). Wischik and colleagues demonstrated that the insoluble PHF consists of a protease-resistant core that retains the characteristic structural features of the intact PHF after Pronase digestion, but lacks a fuzzy outer coat (Wischik et al., 1988a,b; Novak et al., 1993). The tau fragment within the PHF core, corresponds to a sequence of 93–95 amino acids in length that are C-terminally truncated at Glu-391 (Wischik et al., 1988a; Novak et al., 1993). PHFs can be distinguished by their differential solubility. While sarkosyl-soluble PHFs are mainly constituted of tau protein in a hyperphosphorylated state, insoluble PHF fractions from Alzheimer's disease brains are Pronase resistant and phosphorylated tau accounts for no more than 15% of the content of such PHFs (Wischik et al., 1995). Furthermore, rather than promoting the aggregation of tau, phosphorylation of tau is inhibitory for aggregation *in vitro* (Schneider et al., 1999).

The monoclonal antibody (mAb) 423 specifically identifies the C-terminal truncation of tau at Glu-391 in Alzheimer's disease brains (Novak, 1994). We have demonstrated that mAb 423-immunoreactive deposits are present in both intra- and extra-cellular NFTs (Mena et al., 1991, 1995; Garcia-Sierra et al., 2003; Luna-Munoz et al., 2007). Furthermore, C-terminally truncated tau protein has a greater affinity for binding full-length tau molecules (Abraha et al., 2000; Berry et al., 2003) and core PHF-tau over-expressed in transfected COS cells induces apoptosis (Fasulo et al., 1998).

We have combined the techniques of double- and triple- labeled immunohistochemistry with confocal microscopy to examine the molecular changes that arise in pre-tangle cells (Luna-Munoz et al., 2005, 2007). Such cells display the majority of epitopes present in PHFs but in the absence of cytoplasmic fibrillary structures. Examining pre-tangle cells has enabled us to study stages of phosphorylation along the N-portion of tau protein in Alzheimer's disease (Luna-Munoz et al., 2007). In particular, we used four phospho-dependent tau antibodies, namely p-231, TG-3, AT8, and AT100. In addition to mAb 423, TauC3 was used to identify C-terminal truncation of the protein at Asp-421 (Gamblin et al., 2003c; Guillozet-Bongaarts et al., 2005; Luna-Munoz et al., 2007; Basurto-Islas et al., 2008). Early stages of tau processing in pre-tangle cells are characterized by a specific cascade of events in which phosphorylation of the N-terminal domain of tau plays a major role. In addition, we found that Asp-421 truncation at the C-terminus of tau molecule is also present at early stages of tau aggregation (Luna-Munoz et al., 2007).

In this study, we have analyzed the relationship between phosphorylation and truncation in pre-tangle cells. Under normal conditions, truncation at Glu-391 is not detected in aggregates of tau within the cytoplasm of pre-tangle cells. We discuss the consequences of the presence of a highly toxic PHF core in the early stages of tau processing in Alzheimer's disease and discuss how phosphorylated tau may act in a protective manner against the toxicity of the PHF core.

## Materials and methods

### Brain tissue

Brain tissue from six Alzheimer's disease patients (Table 1) was examined in this study (ages, 47–93 years; mean 78.33 years; 2–6 h post-mortem delays) All tissues were obtained from the Brain Bank-LaNSE CINVESTAV-IPN, Mexico in accordance with the institutional bioethics guidelines. The diagnosis of Alzheimer's disease was obtained using the NINCDS-ADRDA group criteria (McKhann et al., 1984), and all samples were Braak stages 5–6. Blocks of hippocampus and adjacent entorhinal cortex were fixed by immersion in a solution of 4% paraformaldehyde in phosphate-buffered saline (PBS), pH 7.4, at 4°C for 7 days.

**Table 1:** **Characteristics of the 6 Brain Samples used in this study**.

**Case #**	**Brain area**	**Sex**	**Age (years)**
1.	Hippocampus CA1	M	80
2.	Hippocampus CA1	F	90
3.	Hippocampus CA1	M	81
4.	Hippocampus CA1	F	47
5.	Hippocampus CA1	F	93
6.	Hippocampus CA1	M	79

*All samples were Braak stages 5–6. Tissues were from the LANSE Brain Bank, CINVESTAV, Mexico City*.

### Antibodies

**Figure 1:**
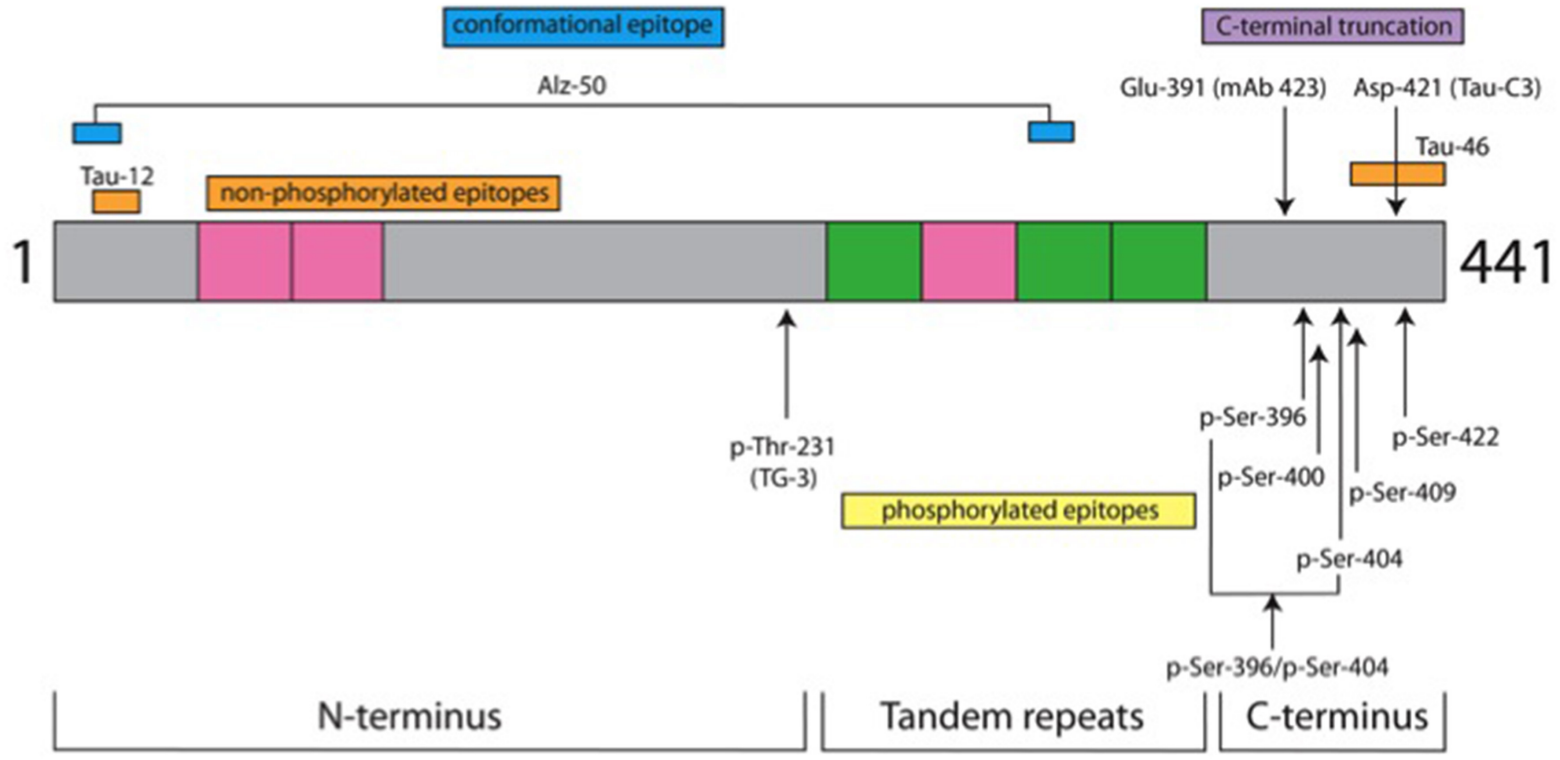
**Tau protein domains and location of antibody epitopes**. The schematic representation shows three domains of tau protein and the location of epitopes for the antibodies used in this study. It is depicted as the longest tau isoform in the central nervous system of 441 amino acids (grey). Inserts from alternatively spliced exons are denoted in pink. There are two in the N-terminus and one in the tandem repeat domain; the other 3 repeat domains are shown in green. Non-phospho-dependent epitopes are shown above the drawing, whereas phospho-dependent epitopes are indicated below. Details for antibodies are listed in Table 2. In green are depicted the repeat domains of tau protein and gray is the tau protein amino acid sequence.

The characteristics of the antibodies used in this study are listed in Table 2 and the location of their epitopes shown in the schematic representation of tau protein in Figure 1.

**Table 2:** **Tau-specific antibodies**.

**Antibody**	**Tau epitope**	**Species/sub-class**	**Dilution**	**References**
**(A) NON-PHOSPHORYLATED EPITOPES**				
Tau-12	N-terminal (residues 9-18)	Mo/IgG	1:1000	(Horowitz et al., 2006)
T46	C-terminal (residues 404-441)	Mo/IgG	1:100	(Preuss et al., 1995)
**(B) CONFORMATIONAL EPITOPE**				
Alz-50	Conformational epitope (residues 5-15 and 312-322)	Mo/IgM	1:500	(Carmel et al., 1996)
**(C) PHOSPHO-DEPENDENT N-TERMINAL EPITOPE**				
TG-3	p-Thr-231	Mo/IgM	1:60	(Jicha et al., 1997b)
**(D) PHOSPHO-DEPENDENT C-TERMINAL EPITOPES**				
pS396	p-Ser-396	Rb/IgG	1:500	(Bramblett et al., 1993)
pS400	p-Ser-400	Rb/IgG	1:100	(Liu et al., 2005)
pS404	p-Ser-404	Rb/IgG	1:100	(Augustinack et al., 2002)
pS409	p-Ser-409	Rb/IgG	1:100	(Jicha et al., 1999b)
AD2	p- Ser-396/p-Ser-404	Mo/IgG	1:500	(Buee-Scherrer et al., 1996)
pS422	p-Ser-422	Rb/IgG	1:1000	(Bussiere et al., 1999)
**(E) C-TERMINAL TRUNCATION EPITOPES**				
TauC3	C-terminal truncation at Asp-421	Mo/IgG	1:1000	(Gamblin et al., 2003c)
mAb 423	C-terminal truncation at Glu-391	Mo/IgG	1:20	(Novak, 1994)

*Abbreviations: Mo, mouse; Rb, rabbit; IgG, immunoglobulin G; IgM, immunoglobulin M*.

### Immunofluorescence

Free-floating sections (50 μm-thick) were exposed to Pronase (0.05% diluted in PBS; Type XIV, Sigma, St Louis, Mo) at 37°C for 30 min, washed with PBS then formic acid (98–100%; Merck, Germany) at room temperature for 3 min. Sections were blocked with a solution of 0.2% IgG-free albumin (Sigma Chemical Co.) in PBS for 20 min at room temperature. Sections were then incubated with the primary antibodies pS396 and mAb 423 (both are IgG subtypes) overnight at 4°C, and then with FITC-tagged goat-anti-rabbit IgG secondary antibody and TRITC-tagged goat-anti-Mouse IgGγ secondary antibody (Jackson ImmunoResearch Laboratories, Inc., West Grove, PA). PBS containing 0.2% Triton X-100 (Sigma Chemical Co.) solution was used in all of the immunolabeling steps for 1 h. Primary antibodies were used at the dilutions indicated in Table 2).

Triple immunolabeling was performed using different combinations. (a) Alz-50 with AD2 and TG3. The corresponding secondary antibodies used were FITC-tagged goat-anti-rabbit IgG, TRITC-tagged goat-anti-mouse IgGγ, and Cy5-tagged goat-anti-mouse IgMμ, respectively. (b) For the combination of TauC3, pS396, and TG-3, FITC-tagged goat-anti-mouse IgGγ, TRITC-tagged goat-anti-rabbit IgG, and Cy5-tagged goat-anti-mouse IgMμ where used. (c) Some sections were immunolabeled with TauC3, AD2, and mAb 423 and counterstained with thiazin red (for the staining of β-pleated structures). These three antibodies are considered to detect late stages of tau processing in NFTs (Galván et al., 2001; Garcia-Sierra et al., 2003, 2008). Control sections were included in which incubations with Pronase and formic acid were omitted. Selected sections were counterstained with thiazin red (TR) to differentiate non-fibrillar from fibrillar states of tau aggregates (Mena et al., 1995, 1996).

### Confocal microscopy

Double and triple immunolabeled sections were mounted with anti-quenching media (Vectashield; Vector Laboratories, Burlingame) and viewed through a confocal laser scanning microscope (TCS-SP2, Leica, Heidelberg) using a 100× oil-immersion plan Apochromat objective (NA 1.4). Ten to fifteen consecutive single sections were sequentially scanned at 0.8–1.0 μm intervals for two or three channels throughout the z-axis of the sample. The resultant stack of images was projected and analyzed onto the two-dimensional plane using a pseudocolor display of green (FITC), red (TRITC) and blue (CY5). Fluorochromes were excited at 488 nm (for FITC), 540 nm (for TRITC) and 650 nm (for CY5).

One hundred images in the areas studied for each combination of antibodies were analyzed (Table 3). From each field in 100× oil objective, we quantified the number of NFTs in both channels and the percentage of colocalization of the signal for all channels. The fields were randomly chosen within the sections. Colocalization analysis was carried out by quantifying the total number of neurons positive for tau protein phosphorylated at S396 and the percentage value of neurons positive to other tau markers, including: Tau-12, T46, TauC3, AD2, and mAb 423, were determined to make 100 visual fields at 100×. The data was normalized to a percentage measure of the number of tau pS396-positive cells that were simultaneously recognized by other tau epitope analyzed.

**Table 3:** **Proportion of NFTs co-labeled with pS396 and other tau antibodies**.

**Antibody combination**	Co-localization of antibodies in NFTs (%)^*^
pS396/Tau-12	7.0
pS396/T46	6.6
pS396/TauC3	47.0
pS396/AD2	83.8
pS396/mAb 423	85.7

* *Co-localization measured for 100 fields at 100× magnification*.

## Results

### Double immunolabeling of NFTs with pS396 and AD2, counterstained with thiazin red

**Figure 2:**
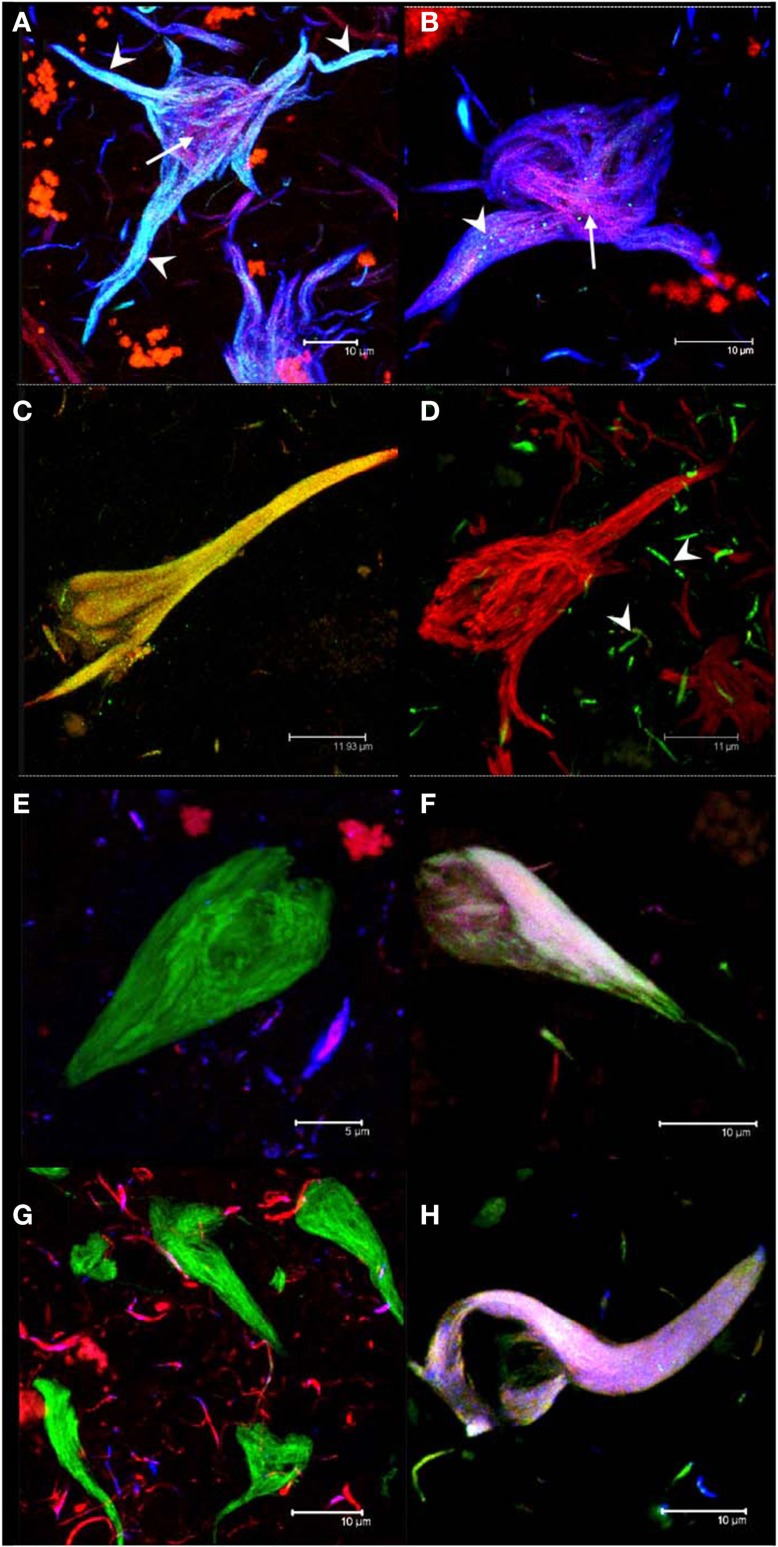
**Double and triple immunolabeling of tangles with antibodies**. Intracellular NFTs were detected by both pS396 and AD2 **(A)**. Extracellular NFTs, however, displayed intense immunoreactivity with pS396 but only sparse and granular staining with AD2 (**B**, arrowheads). Thiazin red detected some PHF bundles located at the center of both intra- and extra-cellular NFTs (**A,B**, arrows). Double immunolabeling with TauC3 and pS396 identified two tangle subtypes: one in which both antibodies were co-localized **(C)** and another that was reactive only with pS396 **(D)**. TauC3 immunoreactivity was restricted to short, thin neurites located in the vicinity of NFTs (arrowheads). Triple immunolabeling with pS396 and Alz-50 with either Tau-12 **(E,F)** or T46 **(G,H)**; and double immunolabeling with pS396 and AD2, counterstained with thiazin red **(A,B)**; and pS396 with TauC3 **(C,D)**. Two subtypes of tangles were observed. (i) The first was recognized by pS396 (green), but not by Alz-50 (blue) either with Tau-12 (red) **(E)** or with T46 (red) **(G)**. (ii) A second subtype was defined as the one which displayed immunoreactivity with all three markers **(F,H)**.

It has been shown that the antibodies AD2 and pS396, having similar epitopes, display a similar pattern of immunoreactivity in tangles (Buee-Scherrer et al., 1996; Galván et al., 2001; Augustinack et al., 2002; Mondragon-Rodriguez et al., 2008). Through double-labeling experiments with AD2 and pS396, we defined two sub-populations of NFTs: one was characterized by the co-localization of pS396 and TR (Figures 2A,B) but lacking AD2 immunolabeling (Figure 2B); the other sub-population of NFTs was distinguished by the co-localization of all three markers (AD2, pS396, and TR). The co-localization of the latter combination of antibodies was strongest in the proximal processes of neurons (Figure 2A; arrowhead). Analysis of pS396 and AD2 double-immunolabeled sections showed that there was co-localization in 84% of tangles (Table 3).

### Double immunolabeling with TauC3 and pS396 in NFTs

Truncation at Asp-421, detected using mAb TauC3, is an early event in tau processing in pre-tangle cells (Rissman et al., 2004; Luna-Munoz et al., 2007; Mena and Luna-muñoz, 2009). In this study, we analyzed the spatial association between pS396 and TauC3 antibodies in NFTs and pre-tangle cells. While the NFT observed in Figure 2C was detected by both antibodies, the tangle observed in Figure 2D was detected only by pS396. Taking into account that the tangle in Figure 2C is intracellular, whereas that in Figure 2D is extracellular, we conclude that the epitope identified by pS396 was more resistant to the extracellular proteolysis than the epitope detected by TauC3. There was 47% of co-localization observed in the NFTs labeled with pS396 and TauC3 (Table 3).

### Triple immunolabeling of NFTs

To determine whether phosphorylation at pS396 was associated with full-length tau, we performed triple immunolabeling with the antibodies for pS396 together with Alz-50 and either Tau-12 or T46 (to non-phosphorylated N- and C- termini, respectively). Alz-50 was included as a marker of conformational changes in the tau molecule. We have previously demonstrated the loss of both C- and N- termini in the protein conformation identified by Alz-50 and that these changes are associated with early events of tau processing present in pre-tangle neuronal cells (Jicha et al., 1997a,b, 1999a; Weaver et al., 2000; Guillozet-Bongaarts et al., 2005; Luna-Munoz et al., 2007). pS396 immunoreactivity (green channel) was present in tangles which were not labeled by Tau-12 (Figure 2E, red channel), T46 (Figure 2G, red channel) or Alz-50 (Figures 2E,G, blue channel). A sub-population of tangles, however, displayed immunoreactivity with all three antibodies (Figures 2F,H). These findings suggest that the epitopes of antibodies Tau-12, T46 and Alz-50 but not that of pS396, are lost during the early stages of tangle formation. For pS396 with AD2 and TauC3, a high level of co-localization in tangles was observed. This was in contrast to the low level of co-localization (6–7%) of pS396 with N- and C-termini (Tau-12 and T46) (Table 3).

### Double immunolabeling with pS396 and mAb 423 in early intracellular tangles

**Figure 3:**
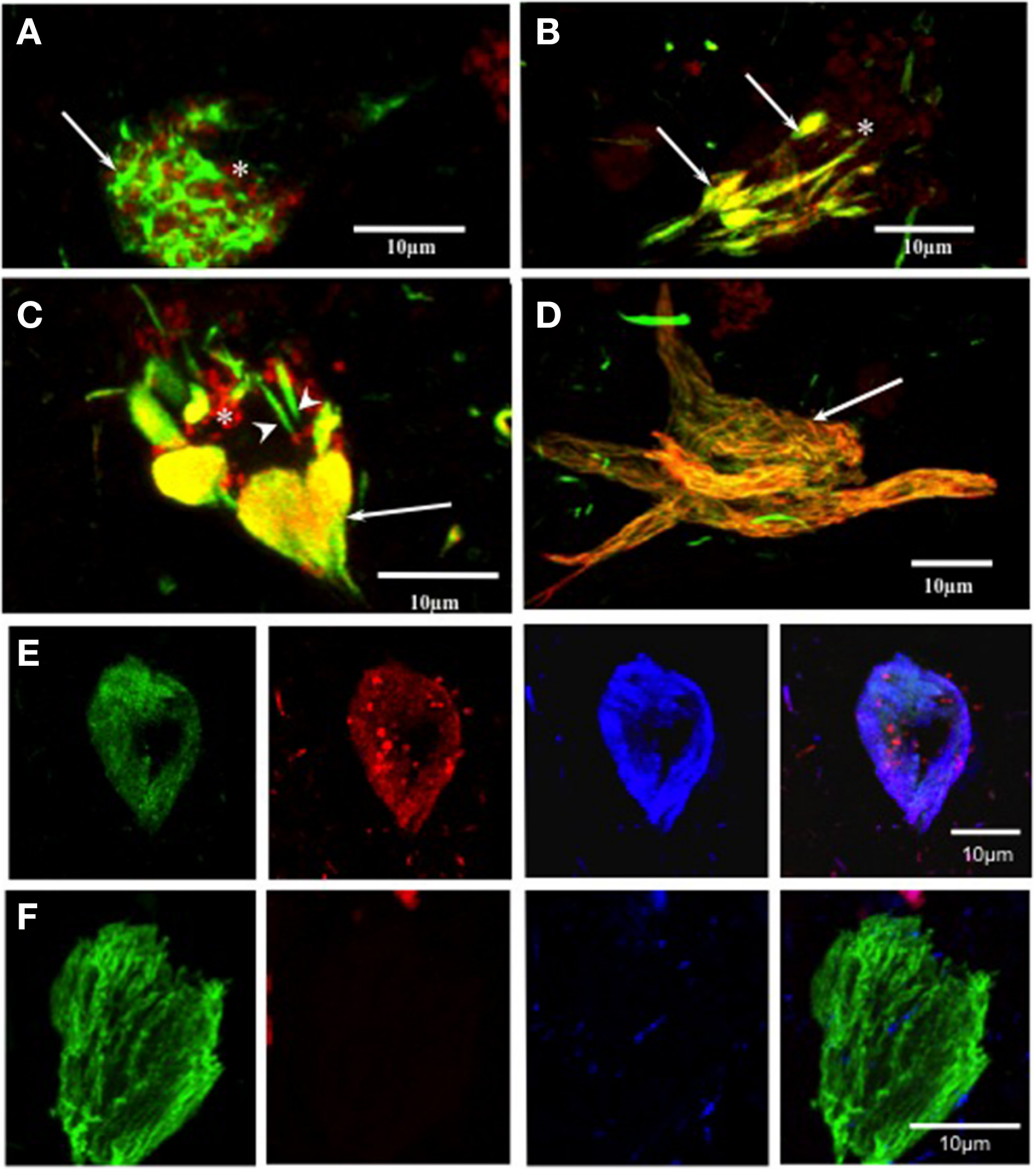
**Double and triple immunolabeling with pS396 and mAb 423, and Alz-50 in neuronal cells with tau degeneration in Alzheimer's disease. (A)** pS396 immunoreactivity was observed in the intracellular early tangles, being present in the perinuclear area (arrows) and closely associated with lipofuscin (arrow, red channel). The mAb 423 failed to identify these structures (red channel, **B, C, D**) In tissue pre-treated with Pronase/formic acid, pS396 and mAb 423 antibodies co-localized in early tangles (**B**, arrows), confluent tangle bundles (**C**, arrows), and NFT (**D**, arrow). **(E, F)** Triple immunolabeling with mAb 423, pS396, and Alz-50 in Pronase/formic acid treated brain tissue, show all three antibodies co-localized in the intracellular tangle **(E)**. An extracellular tangle **(F)** displayed immunoreactivity with only mAb 423. ^*^ lipofuscin granules.

The labeling of intracellular tangles showed co-localization for pS396 and mAb 423 immunoreactivity, as previously described (Garcia-Sierra et al., 2003, 2008). In these early tangles, pS396 immunoreactivity was observed either in an isolated form in the perinuclear area or organized in net-like structures (Figure 3A, arrow). The mAb 423 failed to identify these structures (Figure 3A, arrow).

### Double immunolabeling with pS396 and mAb 423 in Alzheimer's disease tissue pre-treated with pronase/formic acid in early tangles

When brain tissue was treated with Pronase and formic acid prior to immunohistochemistry, mAb 423 immunoreactivity was observed in early tangles and was co-localized with pS396 (Figure 3B, arrows). Clustered, doubly labeled, early tangles were regularly observed. Double immunolabeling was also found in elongated tangle bundles that were associated with auto-fluorescent lipofuscin granules located in the vicinity (Figure 3C, arrow). Some isolated early tangles were detected only by pS396 (Figure 3C, arrowheads). Extracellular tangles were frequently labeled by both pS396 and mAb 423 (Figure 2H, arrow). Eighty-six percent of tangles showed co-localization of these two antibodies (Table 3). The remaining tangles were labeled only by mAb 423, indicating that these were extracellular NFTs.

### Triple immunolabeling with antibodies pS396, mAb 423 and Alz-50 in pronase/formic acid pre-treated brain tissue in NFTs

An intracellular tangle detected by the mAb 423, pS396, and Alz-50 is shown in Figure 3E. There was a trend, however, for the Alz-50 immunoreactivity to be slightly stronger when compared with that of mAb 423 and pS396. Alz-50 and TG-3 are specific markers of intracellular NFTs (Jicha et al., 1997a), and the presence of these two epitopes has been implicated in the early stages of tangle formation (Luna-Munoz et al., 2005). A sub-population of extracellular NFTs was identified by mAb 423 but not by pS396, as illustrated in Figure 3F.

### Triple immunolabeling with antibodies mAb 423, pS396, and Alz-50 in pronase/formic acid-treated brain tissue in pre-tangle cells

**Figure 4:**
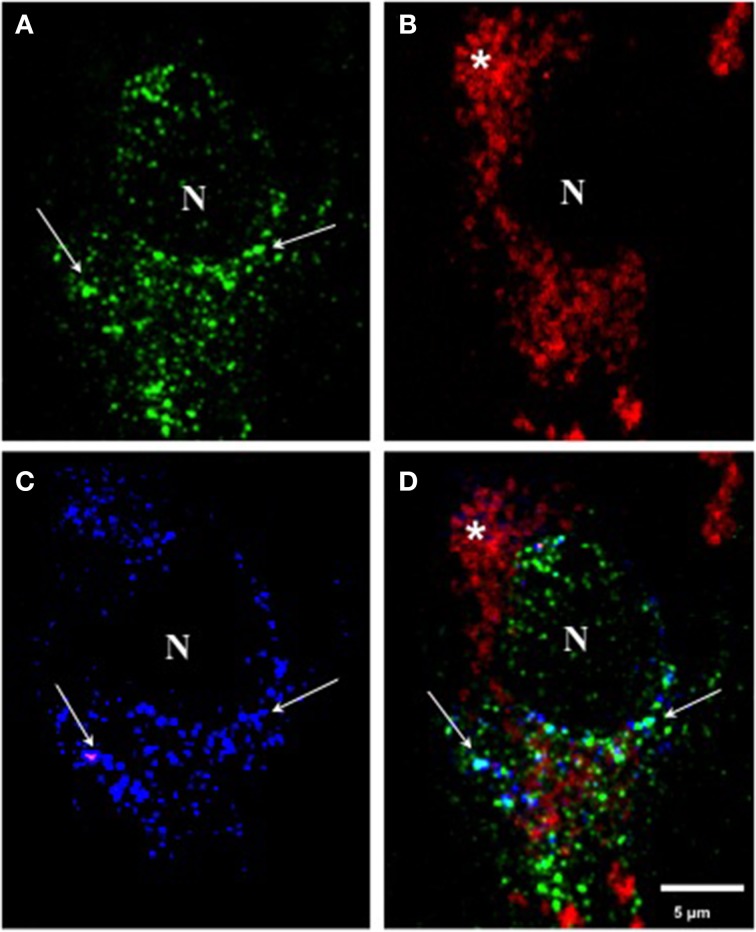
**Triple immunolabeling with antibodies mAb 423, pS396, and Alz-50 of pre-tangle cells after Pronase/formic acid treatment. (A–D)** Triple labeling with mAb 423 (green), Alz-50 (blue) and pS396 (red). A pre-tangle cell displayed diffuse granular cytoplasmic immunolabeling with mAb 423 in the green channel (arrows) the majority of which was also detected by Alz-50 in the blue channel (**B**; arrows). However, pS396 failed to detect such granular structures in the red channel **(B)**. Autofluorescent lipofuscin granules were also observed (^*^). N, nucleus.

When we performed triple immunolabeling with mAb 423, pS396, and Alz-50 in Pronase/formic acid-treated brain tissue, pre-tangle cells, such as the one illustrated in Figure 4, displayed a diffuse granular cytoplasmic immunolabeling with mAb 423 (green channel) and Alz-50 (blue channel), but were not reactive with pS396 (red channel). Although many immunolabeled granular deposits were detected by mAb 423 alone, the majority of such structures that were immunolabeled by Alz-50 were also detected by mAb 423. Some neurons show cytoplasmic clusters of granular appearance.

In early investigations, combining confocal microscopy and electronmicroscopy, we were able to demonstrate that mAb 423 immunoreactivity was present in a diffuse granular pattern in the cytoplasm of putative pre-tangle cells (Mena et al., 1991, 1995), although this immunolabeled pattern has been difficult to find. After the Pronase/formic acid procedure, however, mAb 423 immunoreactivity was revealed in diffuse granular structures in the perinuclear area of the neuronal cell (Figure 4A; arrows). Some of these structures also co-localize with Alz-50 immunoreactivity (Figure 4C; arrows).

## Discussion

The present investigation was focused on the analysis of tau protein processing and aggregation in neuronal soma from the earliest stages (pre-tangle) of granular aggregation prior to the formation of PHFs, which are the components of intracellular and extracellular NFTs. Although NFTs are the structures that best correlate with the evolution of dementia in Alzheimer's disease, we were interested in addressing the early steps involved in their formation.

Our aim was to study the processing and aggregation of tau protein in the neuronal soma at early stages (pre-tangle) to determine the functional role of phosphorylated and truncated tau in the aggregation process at the cellular level.

We previously described that tau protein undergoes a cascade of events in Alzheimer's disease characterized by phosphorylation at specific sites and conformational changes along its N-terminus in pre-tangle neurons (Luna-Munoz et al., 2007). In this report, we have examined early events in tau protein processing along the C-terminal domain of tau and describe that, with the exception of phosphorylation at p-Ser-422, phospho-dependent tau epitopes were not associated with C-terminal truncation at Glu-391. Reactivity with mAb 423 was also absent from these structures. Interestingly, mAb 423 immunoreactivity was evident as granular diffuse deposits in pre-tangle cells after pre-treatment with Pronase/formic acid (Figure 4A), whereas immunoreactivity with pS396, pS400, pS404, and pS409 was absent in these conditions. Immunoreactivity with these C-terminal, phospho-dependent antibodies was observed in structures that represent tau protein aggregation in the pre-tangle stage (Luna-Munoz et al., 2007; Mena and Luna-muñoz, 2009).

**Figure 5:**
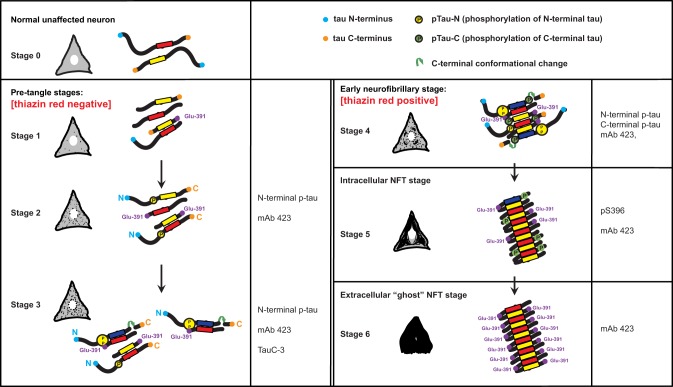
**A model for the stages of tau assembly into PHFs in pre-tangle neurons in Alzheimer's disease**. Unaffected neurons (Stage 0) will have normal tau (neither truncated or hyperphosphorylated) that is associated predominantly with axonal microtubules. Stages 1–3 represent early stages that precede the appearance of fibrillary inclusions. Stage 1 is characterized by the appearance of the PHF core, namely a fragment of 93–95 amino acids in length corresponding to the repeat domain of tau. Stage 2 is defined by the cytoplasmic aggregation of tau molecules resulting from the binding of PHF-core fragments with full-length tau and that is reactive with the N-terminal phospho-dependent antibody. This stage corresponds to the diffuse granular structures seen by confocal microscopy. The PHF-core tau is not evident at this stage, but can be exposed after Pronase/formic acid treatment. Stage 3 corresponds to a further stage in which C-terminal truncation at Asp-421 appears. By Stage 4, intracellular PHFs and fibrils are present, which are phosphorylated at both N- and C-termini and showed variable immunoreactivity to mAb 423. The fibrillar nature of the tangles was confirmed by labeling with TR. As the tangle develops intracellularly in Stage 5, the N-terminal portions are removed and proteolysis reveals the epitope recognized by mAb 423. By Stage 6, the plasma membrane has been disrupted and the extracellular “ghost” tangles are evident, which are comprised of highly insoluble tau, reactive with mAb 423 but with only occasional pS396 epitopes remaining. See text for more detailed description.

The findings described here and in our earlier studies are summarized in a scheme that links the histological observations with the molecular changes in tau protein (Figure 5). Tau protein is predominantly found associated with axonal microtubules in unaffected neurons (Stage 0) but in Alzheimer's disease it accumulates in the somatodendritic compartment of neurons (Binder et al., 1985). The scheme in Figure 5 depicts this process through six stages that are characterized by differential immunoreactivity with antibodies to distinct epitopes along the tau molecule.

The first abnormal event, occurring in the cytoplasm of neuronal cells prone to degeneration in Alzheimer's disease, is characterized by the appearance of the minimal PHF-tau core unit (truncated at Glu-391) within the cytoplasm (Stage 1). This pathological event appears to determine subsequent stages that are characterized by the binding of intact tau molecules to the PHF core. An early series of phosphorylation events, first observed along the N-terminus (Stage 2) (Luna-Munoz et al., 2007; Mena and Luna-muñoz, 2009), would favor the action of caspase-3 that cleaves at Asp-421 (Gamblin et al., 2003c) (Stage 3). These initial steps would go undetected by TR (Uchihara et al., 2001; Luna-Munoz et al., 2007) because the early clusters of both aggregated and less polymerized tau molecules leading to the formation of proto-PHF (tau oligomers of different lengths) (Mena and Luna-muñoz, 2009) are still randomly distributed and do not have fully formed β-pleated sheet conformational structures (Jicha et al., 1997a,b, 1999a; Uchihara et al., 2001). Thus, they have insufficient affinity for the binding of TR (Jicha et al., 1997b; Uchihara et al., 2001; Luna-Munoz et al., 2005). The resultant tau oligomers would act as a template that grows bi-directionally as further tau molecules become sequestered and structurally integrated into the proto-PHF.

According to previous studies of pre-tangles (Mena et al., 1996; Galván et al., 2001; Luna-Munoz et al., 2005), membranous organelles, including mitochondria, nucleus and rough endoplasmic reticulum could serve as a substrate on which the emerging proto-PHF could grow, providing proteolytic stability during elongation of the assembled filaments (Galván et al., 2001). The ability of C-terminally truncated PHF-core tau to capture full-length tau *in vitro* enables sequential cycles of binding, truncation and binding. The origin of this process is not known, but it does not necessarily need to be restricted to a single specific membrane protein alteration; several different proteins or macromolecular complexes could serve as substrates for the initial binding of tau protein. After the initial capture of tau and its proteolysis, the truncated PHF-core tau can bind further tau molecules with increased avidity (Wischik et al., 1996), generating oligomeric aggregates that eventually develop into fibrillary aggregates. Truncation at Glu-391, would confer a special conformation to enable the formation of PHFs, their anti-parallel structural alignment and the restriction of access for the proteolytic enzymes to these oligomers/aggregates/filaments in affected cells (Wischik et al., 1996). Fragments of tau truncated at Glu-391 favor tau polymerization in Alzheimer's disease (Wischik et al., 1996; Berry et al., 2003; Gamblin et al., 2003b). In early tangles, the C-terminal portion of tau would be phosphorylated at Ser-396, Ser-400, Ser-404 and Ser-409 (Stage 4). Interestingly, phosphorylation at Ser-396 seems to be very stable and co-localizes with the PHF core (mAb 423) in the later stages of NFT formation, even after neuronal death (Stages 5 and 6).

The proposed model explaining early mechanisms of tau aggregation in pre-tangle cells, prior to its polymerization into PHFs, deals with two different issues: the toxic and neuroprotective capacities of tau protein (Gamblin et al., 2003a; Castellani et al., 2008; Congdon and Duff, 2008; Garcia-Sierra et al., 2008; Wang and Liu, 2008; Nunomura et al., 2009). Based upon our findings and those from other studies, we propose a model that integrates these processes together with the events of truncation and phosphorylation. We propose that the polymerization of tau protein into PHFs, in Alzheimer's disease, involves complex interactions of truncated and phosphorylated tau species within the cytoplasm of vulnerable neurons (Zilkova et al., 2011). The toxic PHF-core tau fragment would be expected to trigger a cellular protective response, including phosphorylation of normal tau protein and activation of caspases, including caspase-3 (Fasulo et al., 2000, 2005; Gamblin et al., 2003c). Once sequestered in a highly stable and resistant PHF core, full-length tau protein would then become phosphorylated, thereby hiding the toxic species (Bretteville and Planel, 2008; Castellani et al., 2008; Su et al., 2008; Wang and Liu, 2008; Zhang et al., 2009).

This implies that phosphorylation in the early stages of PHF formation would play a protective role, based upon *in vitro* and animal models (Arendt et al., 2003; Stieler et al., 2009, 2011). Moreover tau hyperphosphorylation had been proposed as a protective mechanism, because it is known that this phenomenon could occur in normal (no pathological) conditions, such as during development or hibernation, and in these cases tau hyperphosphorylation does not lead to aggregation or neurodegeneration (Goedert et al., 1993; Arendt et al., 2003; Bretteville and Planel, 2008).

It is important to emphasize that the phosphorylation of the N-terminal portion of the protein tau is a very early event in tau aggregation and abnormal accumulation in Alzheimer's disease (Luna-Munoz et al., 2005, 2007). However, our observations in all Alzheimer's disease cases analyzed here showed that tau phosphorylation at the C-terminus appears to be associated with early stages of tau aggregation when the formation of small TR-reactive tangles occurs. Tau phosphorylation at Ser-422 is observed from the earliest stages of tau aggregation. Previous studies suggested that tau phosphorylation at the Ser-422 can prevent the action of caspase-3, which is very active in Alzheimer's disease and truncates tau at Asp-421 (Guillozet-Bongaarts et al., 2006). Truncated tau is present from early to late stages of tau aggregation. This is why we considered that tau processing and phosphorylation occurs throughout the entire formation and evolution of the NFTs. Two events are relevant for this process: 1) tau processing and 2) sequestration of intact tau and its phosphorylation. This implies that the more internal epitopes of the NFT are the more stable and resistant to proteolysis and not necessarily the last to have been generated. These epitopes could be present throughout the evolution of the NFT, and they become more evident when the NFTs have been treated with Pronase/formic acid, thus exposing the PHF core. In a single tangle, it is possible to find many different tau epitopes corresponding to various stages of processing and phosphorylation of tau protein. Thus, NFTs and pre-tangle structures can be considered as dynamic structures in which multiple pathological processes occur that affect tau protein.

In addition, as proposed by Bondareff and colleagues, a progressive sequence of binding and proteolysis, would eventually provide a template upon which further intact tau molecules can be incorporated into the PHF core and become phosphorylated (Bondareff et al., 1990). Because of the proteolytically resistant conformation adopted by emerging oligomers, all those characterized by Glu-391 truncation would form PHFs. A similar cascade of events will characterize the formation of the NFTs. The predicted effect of both phosphorylation and NFT formation would be the protection of the neuron (Morsch et al., 1999). Eventually, however, the tangled-neuron would die because of abnormal metabolism and/or the release of NFTs into the extracellular space (Mena et al., 1996; Garcia-Sierra et al., 2003; Mena and Luna-muñoz, 2009). The subsequent action of proteolytic enzymes would expose the PHF core as the ghost or extracellular NFT appears (Garcia-Sierra et al., 2003, 2008; Guillozet-Bongaarts et al., 2005). It remains to be determined whether NFTs, purified from Alzheimer's disease brains, are toxic. These studies, however, provide important insight into the characterization of tau aggregates as they exist *in vivo*.

### Conflict of interest statement

The authors declare that the research was conducted in the absence of any commercial or financial relationships that could be construed as a potential conflict of interest.
